# The Editor's Letter-Box

**Published:** 1918-08-24

**Authors:** Lynette Gould

**Affiliations:** Sister-In-Charge, Preliminary Training-School, King Edward VII.'s Hospital, Cardiff. Anthony House, Cardiff


*To the Editor of* THE HOSPITAL.

SIR,—The loyalty that is felt for their Alma Mater by "Old Londoners" of the first rank is quite as strong and true among those of a younger generation and occupying less distinguished posts. Indeed, so strong is it that it seems hardly worth while to try and disturb it in these days of scanty leisure and paper shortage!—I am, yours faithfully, LYNETTE GOULD,

Sister-in-Charge, Preliminary Training-School,
King Edward VII.'s Hospital, Cardiff.

Anthony House, Cardiff.
August 18.

